# Prevention of Spinal Cord Injury during Thoracoabdominal Aortic Aneurysms Repair: What the Anaesthesiologist Should Know

**DOI:** 10.3390/jpm12101629

**Published:** 2022-10-01

**Authors:** Federico Marturano, Fulvio Nisi, Enrico Giustiniano, Francesco Benedetto, Federico Piccioni, Umberto Ripani

**Affiliations:** 1 Department of Anaesthesia, Analgesia and Intensive Care, Vito Fazzi Hospital, 73100 Lecce, Italy; 2 Department of Anaesthesia and Intensive Care Unit, IRCCS Humanitas Research Hospital, 20089 Milan, Italy; 3 Department of Biomedical Sciences, Humanitas University, 20090 Milan, Italy; 4 Division of Clinic Anaesthesia, Department of Emergency Hospital Riuniti, Conca Street 71, 60126 Ancona, Italy

**Keywords:** aortic repair, aneurysm, vascular surgery, neuroprotection, neuromonitoring, CSF drainage

## Abstract

Thoraco-abdominal aortic repair is a high-risk surgery for both mortality and morbidity. A major complication is paraplegia-paralysis due to spinal cord injury. Modern thoracic and abdominal aortic aneurysm repair techniques involve multiple strategies to reduce the risk of spinal cord ischemia during and after surgery. These include both surgical and anaesthesiologic approaches to optimize spinal cord perfusion by staging the procedure, guaranteeing perfusion of the distal aorta through various techniques (left atrium–left femoral artery by-pass) by pharmacological and monitoring interventions or by maximizing oxygen delivery and inducing spinal cord hypothermia. Lumbar CSF drainage alone or in combination with other techniques remains one of the most used and effective strategies. This narrative review overviews the current techniques to prevent or avoid spinal cord injury during thoracoabdominal aortic aneurysms repair.

## 1. Introduction

Thoracic endovascular aortic repair (TEVAR) is a minimally invasive option for the management of the pathology of the descending thoracic aorta compared to open surgical repair (OPEN). Mortality of OPEN surgery for thoracic aortic aneurysm (TAA) is between 3 and 28%, while for endovascular correction it is between 0 and 16.9% [1]. TEVAR reduces the morbidity and mortality rates compared to the OPEN procedure but is associated with a significant risk of spinal cord ischemia (SCI) and consequently paraplegia (22% open versus 19% endovascular) [2]. This risk is similar between the two procedures [3]. Spinal cord blood flow depends on a single anterior longitudinal spinal artery and two posterolateral spinal arteries, which form a plexus running along the medulla. Anterior and posterior spinal arteries originate from vertebral arteries and receive a contribution from segmental arteries, collateral vessels emerging directly from the aorta or left subclavian artery [4]. In the thoracic region, arterial blood flow is supplied directly by the aorta through intercostal arteries; the Adamkiewicz artery, also known as arteria radicularis magna, is a large radicular artery that originates between T8 and T12 and is the most important collateral artery at the thoracic level [5]. At the lumbar and sacral level, collateral circles are provided by lumbar arteries (parietal branches of the abdominal aorta) and by the lateral sacral arteries (parietal branches of the internal iliac artery).

## 2. Pathophysiology of Spinal Cord Ischemia

The pathogenesis of spinal cord ischemia after TEVAR is multifactorial and is not completely elucidated. In the past, Adamkiewicz artery’s occlusion was supposed to be a main contributor of spinal cord ischemia after aortic aneurysm repair [6], but current data show alterations of collateral circulation have greater relevance in the development of SCI [7]. Two theories have been developed in this regard: the first is based on the inadequate remodelling of collateral vessels’ network; the latter involves micro embolism originated from aortic plaques in segmental arteries. Collateral circulations are a network of blood vessels interconnecting the anterior spinal artery with vessels that supply blood to the adjacent muscles of the back [4]. These interconnections act as an important alternative supply of blood when the main sources are excluded, such as during aortic clamping in open repair or exclusion of segmental arteries after release of the thoracic stent [8]. These features interact with the hydrodynamics of the cerebrospinal fluid. In OPEN repair of TAA, aortic clamping causes systemic arterial hypertension proximal to clamping and hypotension distal to the clamp and increased splanchnic and central venous pressure [9] raising intracranial pressure (PIC) and cerebrospinal fluid (CSF) pressure [10]. Distal systemic arterial hypotension causes renal, intestinal, and lower limb ischemia and reduces spine cord perfusion pressure (SCPP) if we consider the main vessels which guarantee the blood perfusion supply, according to the following relationship [11]:

SCPP = MAPd (mean arterial pressure distal to clamp) − CSF pressure

Furthermore, after aortic unclamping, reperfusion may contribute to spinal cord injury as well, through inflammatory response, hypercarbia, which increases CSF pressure, and systemic hypotension which decreases spinal perfusion pressure. Metabolic acidosis can increase cerebral blood flow, intracranial pressure, and CSF pressure upon unclamping. Moreover, anaerobic metabolites are responsible for a reduction in peripheral vascular resistance and therefore for severe hypotension. Spinal cord oedema caused by reperfusion injury can also increase CSF pressure [12]. During TEVAR, the remodeling of the collateral network plays a fundamental role in maintaining spinal perfusion through changes in distribution of blood flow between intraspinous and paraspinous collateral network after the exclusion of segmental arteries [13]. These changes occur between the second and fifth day after surgery [13]. Furthermore, non-occluded segmental arteries can ensure perfusion to ischemic spinal segments by reversing the direction of blood flow in the anterior spinal artery through proximal and distal segmental arteries. SCI may arise if all these compensatory mechanisms fail or if supplying vessels are obstructed by thrombo-embolic formations or by flow alterations due to the stent [14]. Collateral circles that supply blood to the spine are the left subclavian artery, intercostal, lumbar, and hypogastric arteries. There is a close correlation between the coverage of two or more collateral circles and the development of symptomatic medullary ischemia, especially when associated with prolonged perioperative hypotension. The left subclavian artery is one of the most important tributary arteries of collateral circulation to the spine. Indeed, it ensures blood flow both to the brain and spinal cord through the left vertebral artery, which divides into the basilar artery, perfusing the posterior part of the circle of Willis and into the anterior spinal artery directed to the spinal cord. In addition, the thyrocervical trunk arising from the left subclavian artery supplies blood to the spine through anterior and posterior radicular arteries. Unfortunately, more than 40% of patients undergoing TEVAR require intentional coverage of the left subclavian due to the proximity of thoracic aortic disease [15]. This increases the risk of spinal cord injury. The internal iliac artery, also known as hypogastric, supplies blood to the spine through lumbosacral radicular arteries [15]. During TEVAR, impaired hypogastric blood flow may occur when an iliac artery graft is used as a vascular access in patients with severe atherosclerosis of the ileo-femoral arteries. This was associated with a high risk of spinal cord ischemia (4.3%) [16]. The extension of the aortic coverage by stents is one of the most important surgical risk factors which increase the risk of SCI by 30% every 2 cm of additional aortic coverage [17].

## 3. Clinical Presentation

The clinical presentation of SCI varies from limb paresis to complete paraplegia with or without autonomic dysfunction. These manifestations can be transient with partial or complete recovery or permanent [18]. SCI can manifest immediately after surgery, the patient showing focal neurological deficits upon awakening from anaesthesia or more often with a delayed onset, 24–48 h after surgery [5,19]. Early onset SCI is associated with a negative prognosis since it is not possible to determine whether the onset of ischemia and spine cord infarction has already begun. On the other hand, delayed onset SCI, when promptly detected and treated, has a relatively good prognosis [20,21]. Immediate paralysis occurs more frequently after OPEN repair, whilst delayed onset paralysis occurs generally after TEVAR. The different clinical presentation during OPEN repair is due to clamping that quickly avoids perfusion to intercostal arteries suppling blood to anterior part of spinal cord. After unclamping, the blood flow to these arteries is restored. The ischemia-reperfusion damage caused by clamping–unclamping contributes to immediate SCI. On the other hand, during TEVAR the stent permanently covers the intercostal arteries branches with a constant reduction of blow flow and the development of compensatory circulations. This avoids the risks of reperfusion damage but induces long-term effects of impaired spinal blood flow compared to the shorter period of aortic clamping during OPEN repair [22]. Tthe ideal preventive and treatment measures for spinal cord ischemia after TEVAR are still a matter of debate, but most strategies are aimed either at increasing mean arterial pressure or at reducing the cerebrospinal fluid pressure through CSF drainage to optimize medullary perfusion. Current guidelines [23] recommend CSF drainage for spinal cord protection in both open and endovascular surgery in patients at high risk of SCI. Risk factors for development of SCI after thoracic aortic repair are both related to the patient and associated with the surgical procedure. They are listed in Table 1.

## 4. Neuroprotective Strategies for Spinal Cord during TEVAR

Avoiding SCI during thoracic aortic aneurysms repair is essential. Several strategies can be implemented to prevent SCI, both surgical and anaesthetic (Table 2).

### 4.1. Surgical Strategies

In cases where an extensive coverage of the aorta is planned (e.g., >30 cm), a two-stage procedure can be considered to reduce risk of SCI [24]. A two-stage approach includes firstly implanting a thoracic proximal endograft above emerging vessels. After two to three months, a distal stent will be placed [25]. Furthermore, surgical options to ensure flow to the left subclavian artery are revascularization or endovascular techniques are used. Revascularization procedures include the transposition of the subclavian on the left common carotid artery or insertion of a small bypass [26]. Endovascular techniques involve the use of fenestrated/branched stents, chimney and periscope graft, sandwich techniques, and fenestration techniques in situ [27]. Current guidelines [28] suggest previous revascularization of the left subclavian artery in patients undergoing TEVAR if proximal stent seal covers the artery emergence. Selective revascularization is absolutely indicated in patients with coronary artery bypass grafted with internal mammary artery in the presence of an arteriovenous fistula on the left arm and in left-handed patients. Relative indications include dominance of the left vertebral artery, extensive aortic coverage, previous AAA repair, and occlusion of the hypogastric artery. Post-operative indications to revascularization concern upper limb ischemia or onset of vertebrobasilar insufficiency [29]. Minimally invasive segmental artery coil embolization (MISACE) is a novel technique preconditioning to ischemia, inducing neo-angiogenesis, and improving vascularization of spinal cord before TEVAR. Despite encouraging prospects, reproducibility of this technique in patients with tortuous vessels or with thrombi localized in the aneurysm sac is not easy [30], and there are also concerns because the procedure itself can cause SCI. Temporary aneurysm sac perfusion (TASP) is a technique involving a two-stage procedure: the first step consists of the creation of an endoleak in the lateral branch of the endoprosthesis which perfuses the main splanchnic vessels, and one to three months later, a stent deployment will close the endoleak [31]. The use of branched or fenestrated stents is associated with a lower risk of the onset of SCI [32].

### 4.2. Anaesthesiologic Strategies

The anaesthetic approach to perioperative management of thoracic aortic aneurysms repair is fundamental and contributes to the prevention and management of complications. A goal-directed hemodynamic strategy is mandatory to achieve adequate spinal cord oxygenation and perfusion by a correct and adequate management of cardiac index (>2.5 L/min/m^2^) and serum hemoglobinemia concentration [18]. As stated before, spinal cord perfusion pressure is the difference between mean arterial pressure (MAP) and CSF pressure [10]. Optimizing systemic arterial blood pressure (MAP > 90 mmHg) and CSF pressure (<10 mmHg) allows to achieve an adequate spinal cord perfusion pressure (>80 mmHg) [33]. Data reported in literature [18] demonstrates that maintenance of spinal cord perfusion pressure >80 mmHg through MAP increase and CSF drainage after placement of the aortic stent can prevent and reverse SCI and paralysis. In patients developing postoperative ischaemia, MAP > 90 mmHg should be the first step of treatment (vasoactive drugs). Vasopressors should be gradually reduced over the next 24–48 h after the improvement of symptoms [34]. Neurophysiological monitoring is useful to detect spinal cord ischemia during both open and endovascular surgery [22,33]. Motor evoked potentials (MEPs) can evaluate descending spinal pathways, while somatosensory evoked potentials (SSEP) are used for ascending pathways. One limitation to their application is the inability to differentiate medium from severe SCI. They are also affected by lower limb ischemia caused by vascular introducers [35]. Furthermore, volatile anaesthetics alter cortical waves, increasing latency and reducing the amplitude of SSEP; then, they should be administered at no more than 0.5 MAC [36,37]. For these reasons, intravenous anaesthesia is recommended when evoked potentials are monitored (e.g., propofol and remifentanil infusions and, if necessary, low concentrations of volatile anaesthetic) [38]. Administration of neuromuscular blockers must also be carefully monitored to ensure muscle relaxation and an adequate MEP response. Near infrared spectroscopy (NIRS) is another useful monitoring used during TEVAR for diagnosis of SCI. Electrodes are placed on the surface of paraspinous muscles at the thoracic and lumbar level. NIRS provides measurement of blood oxygen saturation in paraspinous circulations, close to spinal cord microcirculation, reflecting indirectly its oxygenation and blood perfusion [39]. However, this promising technique has yet to be clinically validated [40], and its routinary use is not yet recommended [41]. Moderate hypothermia (32–35 °C) may play a role in spinal cord neuroprotection [42]. Hypothermia may be used to provide some degree of acute tolerance of spinal cord to perfusion disruption during surgery, especially OPEN. It can be achieved systemically through cardiopulmonary bypass or by infusion of cold fluids into epidural space [41]. However, systemic hypothermia is associated with several risks, such as dysrhythmias, coagulopathy, and metabolic alterations, while selective spinal cord hypothermia through epidural cooling is an invasive procedure, applicable for a limited duration with high risk of contamination and spinal cord rebound oedema [43]. However, this practice is poorly used in TEVAR as it requires a very invasive approach compared to the procedure which is minimally invasive [44], and in vitro studies showed hypothermia can alter the structure of grafts, causing deformation, migration, and endoleak [45]. The pharmacological protective strategy can also reduce spinal cord metabolic demands and inflammatory-neurochemical response to ischemia and reperfusion, especially when combined with other preventive strategies, such as CSF drainage. High dose of methylprednisolone (30 mg/kg) before aortic clamping [46] or intrathecal papaverine to improve spinal cord perfusion [47] or naloxone infused at a rate of 1 mcg/kg/h, starting before induction up to 48 h after surgery [48], are the most adopted strategies. SCI-induced neuronal cell death is triggered by the presence of redundant excitatory neurotransmitters, especially glutamate, glycerine, and aspartate that cause an excessive influx of calcium [49]. In experimental settings, naloxone reduces CSF glutamate and aspartate concentrations but not glycerine [50]. However, today there are no definite recommendations on routine use of these drugs to avoid SCI. CSF drainage is the most effective procedure in preventing SCI after aortic repair because acute changes due to ischemia and reperfusion result in spinal cord oedema and increased CSF pressure and finally SCPP reduces. Current guidelines [22] recommend CSF drainage for both open and endovascular TAA repair in patients at high risk of SCI. Specific indications to CSF drainage in patient undergoing TEVAR were recently identified (Table 3), although further validation needs [21]. 

Postoperative CSF drainage is indicated in patients with delayed paralysis following TEVAR within two hours of symptom onset. Although recent works [18] do not recommend this practice since the mechanisms of delayed paraplegia are not fully understood yet, CSF drainage can be performed on the day before surgery to recognize early any sort of complications [51] or can be scheduled on the day of surgery [40]. In any case, the timing of CSF drain placement does not have an impact on the post-discharge functional impairment or long-term mortality [52]. Hemodynamic optimization plus CSF drainage is currently recommended as the best strategy to avoid or reduce risks of SCI during TEVAR [40]. For this purpose, a silicon drainage catheter is inserted approximately 8–10 cm into the subarachnoid space at the lumbar level, allowing measurement of CSF pressure and CSF drainage [53]. Current recommendations [22,23,24,25,26,27,28,29,30,31,32,33,34,35,36,37,38,39,40,41,42,43,44,45,46,47,48,49,50,51,52,53,54,55] suggest continuous monitoring of CSF pressure to monitor the ICP wave and prevent catheter obstruction. Intermittent rather than continuous CSF drainage reduce the risks of the development subarachnoid haemorrhage due to excessive loss of CSF in a short time [56,57,58]. The rate of complications after lumbar drainage placement is 0.3–1.0% [59]. The most frequent is localized infection (11.1%), post-dural puncture headache (3.3%), puncture site bleeding (2.1%), persistent CSF loss (1.3%), subdural hematoma with no clinical evidence, abducens nerve palsy, catheter displacement-occlusion (0.1%), or fracture (0.15%). More severe complications are meningitis (0.1%), subdural hematoma (1.7%), and intracranial haemorrhage (1.8%) [60]. The latter may be associated with excessive CSF drainage stretching and tearing of the dural veins. To minimize the risks of subdural hematoma, a CSF pressure ≥10 mmHg is recommended in the absence of ischaemia or to perform an intermittent drainage of 10–20 mL/h with continuous blood pressure monitoring. Patients with cerebral atrophy, arteriovenous malformations, brain aneurysms, and history of previous subdural hematoma are particularly disposed to develop cerebral haemorrhage [57]. Normal haemostatic panel and platelet count is also recommended before the procedure and withdrawal time of any anticoagulant and antiaggregant drugs must be properly respected [61]. The catheter should be kept in place for at least three days. In fact, delayed paralysis occurs after 1.8 days and is frequently associated with hypotension or catheter malfunction. CSF aspiration of 10–20 mL/h is recommended if PIC > 10 mmHg, while fluid or vasopressor infusion can support the hemodynamics [62].

In addition to this, CSF drainage system may be useful to measure the lactate concentration in the cerebrospinal fluid [63]. During descending aorta surgery, CSF lactate has been proposed as a marker of neuronal anaerobic metabolism due to spinal ischemia related to aortic clamping proximal to the Adamkiewicz arteria or to the exclusion of the main spinal arteries from the blood flow because of the endovascular prosthesis, and its increase occurs earlier than other markers, such as S-100 protein [64,65]. Noteworthy, in neurosurgical patients, point-of-care measurements of cerebrospinal fluid lactate could aid in detecting infections of the central nervous system and surrounding structures [66]. This implies that CSF-lactate levels may even increase for reasons different from spinal cord ischemia [67]. Nevertheless, Casiraghi et al. demonstrated that preoperative baseline CSF lactate levels were somehow higher in patients with spinal cord injury compared with those with no injury [68]. Normal values of spinal fluid lactate concentration are debated. According to some authors, normal concentration is age-related and ranges from 0.6 to 3.1 mmol/L, with increasing levels which also depend on the duration of the low-flow state when spinal ischemia occurs [63].

#### Type of Anaesthesia

General anaesthesia is mandatory for open thoracic aortic surgery, and it is often performed for TEVAR. This allows a safe control of the airways in case of haemorrhagic shock or rapid conversion to open surgery, the possibility for intraoperative monitoring with trans-esophageal echocardiography, avoids involuntary movements of the surgical field related to the patient’s respiratory pattern, and ensures the pacemaker’s heart rate control. Nevertheless, neuraxial and locoregional anaesthesia are emerging techniques in TEVAR [69]. Neuraxial anaesthesia can provide adequate pain relief on large bore of vascular access sites and relieve discomfort related to the prolonged fixed position. Moreover, it allows continuous monitoring of clinical signs of cerebral ischemia. On the other hands, spinal anaesthesia is associated with haemodynamic instability and with the risk of spinal hematoma. Local anaesthetic infiltration of femoral access sites, combined with slight sedation (also known as monitored anaesthesia care), is an alternative technique preserving patient’s consciousness and ability to obey orders without pain and haemodynamic stability and finally allowing to directly monitor the spinal cord function. In these cases, patient cooperation is crucial as spontaneous movements are possible during critical surgical manoeuvres, and pain and stress are less manageable. Currently, there are insufficient scientific data to recommend any of these techniques as a first choice in the management of TEVAR; still evidences in endovascular abdominal aneurysm repair (EVAR) show less postoperative pulmonary complications [70], shorter in-hospital length of stay, and lower mortality [71] in patients undergoing regional/local anaesthesia.

### 4.3. Special Consideration for Aortic Dissection Repair

Aortic dissection is a life-threatening condition caused by tears of the aortic inner layer through which blood enters and flows. The resulting false lumen can lead to occlusion of aortic branches and consequent ischemia. The Stanford classification divides aortic dissections into type-A with involvement of the ascending aorta and type-B, originating distal to the left subclavian artery. Emergency surgery is mandatory for treatment of type-A dissections. Total arch replacement is obtained by a Dacron prosthesis (frozen elephant trunk) that provides an anchoring platform for following distal endograft.

Spinal cord injury is a well-known complication following this procedure (8.9%) [72].

Risk factors associated with SCI are stent extent greater than 15 cm, a coverage at or distal to T8, and length of circulation arrest [73]. Ensuring blood flow to the left subclavian and femoral artery via extracorporeal circulation during circulation arrest and guaranteeing hypothermia are techniques used to prevent SCI during aortic arch repair [74]. Intraoperative CSF drainage is not currently recommended due to emergency surgery, but it can be useful postoperatively to treat sudden paraplegia, according to data of aortic aneurysm repair [75]. Not-complicated type-B dissection requires a first step conservative treatment with medical therapy and rest. Then, endovascular surgery can be planned. Urgent TEVAR is indicated in the type-B dissection presenting with persistent pain, uncontrolled hypertension, early aortic expansion, signs of malperfusion, or rupture [76]. In all these cases, recommendations similar to those postulated for aneurysm repair can be applied for the prevention of SCI.

## 5. Conclusions

Thoracic aneurysm repair is associated with high risks of perioperative complications. Spinal cord ischemia injury is the most frequent complication causing paraplegia. Each case scheduled for surgery must be discussed between surgeons and anaesthesiologists to consider case-by-case benefits and risks related to thoracic aneurysm repair. Several strategies can be implemented to avoid or reduce the risk of SCI. Anaesthesiologists should guarantee adequate spinal cord perfusion by increasing MAP to ensure collateral circulation and decreasing CSF pressure through CSF drainage. All the other interventions still need a prospective validation, and their uses should be tailored individually on to the patient.

## Figures and Tables

**Table 1:** Risk factors for susceptibility of spine cord ischemia after thoracic aortic repair ^1^.

Patient Risk Factors	Surgical Risk Factors
1-Advanced age (>70 yrs.)	1-Total aortic coverage > 205 mm
2-Perioperative hypotension (MAP < 70 mmHg)	2-Concomitant abdominal aortic aneurysm or prior abdominal aortic aneurysm surgical repair
3-Renal insufficiency (Creatinine > 132 μMol/L)	3-Coverage of ≥2 vascular territories
4-COPD	4-Left subclavian artery coverage
5-Hypertension	5-Urgent procedure
6-Degenerative aneurysm	6-Coverage of hypogastric artery
	7-Adjunct procedure (Iliac conduit)
	8-Use of ≥3 stents
	9-Longer duration of the procedure
	10- Excessive blood loss

^1^ Adapted from [22] Hiratzka LF, Bakris GL, Beckman JA, et al. 2010 ACCF/AHA/AATS/ACR/ASA/ SCA/SCAI/SIR/ STS/ SVM guidelines for the diagnosis and management of patients with thoracic aortic disease: a report of the American College of Cardiology Foundation/American Heart Association Task Force on Practice Guidelines, American Association for Thoracic Surgery, American College of Radiology, American Stroke Association, Society of Cardiovascular Anesthesiologists, Society for Cardiovascular Angiography and Interventions, Society of Interventional Radiology, Society of Thoracic Surgeons, and Society for Vascular Medicine. *Circulation* **2010**, *121*, 266–369.

**Table 2:** Anaesthetic and surgical strategies to prevent and treat spine cord ischemia.

Anaesthetic Approaches	Surgical Techniques
1-Maximize oxygen delivery (increase Cardiac Index > 2.5 L/min/m^2^ or optimize haemoglobin)	1-Staging the procedure
2-Mild hypothermia (32–35 °C)	2-Left subclavian artery revascularization
3-Maintain CSF pressure (≤10 mmHg)	3-Minimally invasive segmental artery coil embolization (MISACE)
4-Maintain spinal cord perfusion pressure (≥80 mmHg)	4-Temporary aneurysm sac perfusion (TASP)
5-Neuromonitoring (e.g., motor/sensory evoked potentials, NIRS)	5-New implantation sequence for branched/fenestrated stents
6-Monitor CSF lactate	

**Table 3:** Indications for positioning CSF drainage in patient undergoing TEVAR ^1^.

Indications for the Use of CSF Drain in TEVAR
1. Anticipated endograft coverage of T8-L1 (especially intercostal/lumbar arteries that supply Adamkiewicz artery identified by preoperative CT/MRI angiography).
2. Coverage of a long segment of thoracic aorta (>30 cm).
3. Compromised collateral pathways (e.g., previous infrarenal aortic aneurysm repair, occluded hypogastric arteries, coverage of the left subclavian artery without revascularization).
4. Symptomatic spinal ischemia in a patient who did not have a drain placed preoperatively.

^1^ Adapted from [23] Uchida N. How to prevent spinal cord injury during endovascular repair of thoracic aortic disease. *Gen Thorac Cardiovasc Surg* **2014**, *62*, 391–397.
